# Postoperative dendritic cell vaccination in brain metastasis: A real-world retrospective cohort study evaluating survival and safety

**DOI:** 10.1097/MD.0000000000045178

**Published:** 2025-10-10

**Authors:** Chih-Hsiu Tu, Wei-Lin Hsu, Chun-Jen Chang, Yu-Chung Juan, Wen-Liang Huang, Ming-Chao Liu, Yu-Han Huang, Huai-Ping Ho, Hung-Lin Lin, Jeng-Hung Guo, XianXiu Chen, Der-Yang Cho, Chun-Chung Chen

**Affiliations:** a Department of Neurosurgery, China Medical University Hospital, China Medical University, Taichung, Taiwan; b Department of Internal Medicine, China Medical University Hospital, China Medical University, Taichung, Taiwan; c Graduate Institute of Biomedical Sciences, College of Medicine, China Medical University, Taichung, Taiwan; d Department of Medical Research, Translational Cell Therapy Center, China Medical University Hospital, Taichung, Taiwan; e Neuroscience and Brain Disease Center, China Medical University, Taichung, Taiwan; f Graduate Institute of Acupuncture Science, College of Chinese Medicine, China Medical University, Taichung, Taiwan.

**Keywords:** brain metastasis, brain tumor, dendritic cell, immunotherapy, overall survival, vaccine

## Abstract

Brain metastasis (BM) is associated with poor prognosis and limited therapeutic options. Immunotherapy with dendritic cell (DC) vaccination represents a promising approach by potentially overcoming blood-brain barrier limitations while generating tumor-specific immune responses. This study evaluates the safety and preliminary efficacy of postoperative DC vaccination in patients with surgically resected BMs. We conducted a retrospective analysis of 17 patients (9 women, 8 men; mean age: 56.2 years) with BM from diverse primary cancers who received conventional treatment plus autologous DC vaccination between 2019 and 2022 at a single institution. Following tumor resection, patients received radiotherapy and/or systemic therapy alongside DC vaccination (2 × 10^8^ DCs administered subcutaneously) over 6 months. Patients were assessed for adverse events, overall survival (OS), and survival relative to prognostic expectations based on diagnosis-specific graded prognostic assessment (DS-GPA). The median OS was 18 months (95% CI: 9–22), compared to historical median of 14.5 months for surgical resection alone. Patients receiving ≥ 6 vaccine doses demonstrated a median OS of 18 months (95% CI: 9–24) versus17 (95% CI: 3–24) for those receiving < 6 doses (Log-rank test, *P* = .39). Among 14 patients with available DS-GPA scores, 9 (64.3%) survived longer than their predicted median survival. No vaccine-related serious adverse events were observed, with reactions limited to grade 1 injection site reactions and low-grade fever. This real-world analysis suggests that postoperative DC vaccination for BM is safe and may confer survival benefit compared to historical outcomes. While limited by sample size and retrospective design, our findings warrant further investigation in prospective controlled trials to definitively establish efficacy and optimal integration into multimodal treatment approaches.

## 1. Introduction

Brain metastases (BMs) are 10 times more common than primary brain tumors,^[1]^ with their incidence steadily increasing due to improved diagnostic capabilities and longer survival of cancer patients.^[2]^ Approximately 50% of patients with cancer experience metastasis to the central nervous system (CNS) during the course of their disease.^[3–5]^ Lung cancer, breast cancer, and melanoma are the 3 primary cancers that account for over 75% of BMs.^[6,7]^ The prognosis for patients with BMs remains poor, with estimated 2-year and 5-year overall survival (OS) rates of only 8.1% and 2.4%, respectively.^[1]^ Metastasis to the CNS causes death in over half of these patients, highlighting the urgent need for more effective therapeutic approaches.¹ Even with current standard management, the median OS of patients who undergo surgical resection of BMs is only 14.5 months.^[8]^

Current therapeutic strategies for patients with BM primarily focus on local disease control with palliative intent, including surgical excision, whole-brain radiotherapy, stereotactic radiosurgery, or combinations of these approaches.^[4]^ Systemic therapies have historically played a limited role due to the blood-brain barrier (BBB), which restricts the penetration of many therapeutic agents into the CNS.^[1]^ This has created a significant therapeutic challenge, as patients with BM often experience progression despite aggressive local therapies.

While the CNS was traditionally considered immune-privileged due to the BBB, emerging evidence suggests that immune cells can indeed infiltrate the CNS under pathological conditions, creating opportunities for immunotherapeutic interventions.^[9,10]^ Immune checkpoint inhibitors (ICIs), such as anti-PD-1 antibodies, have shown promising results in the treatment of BMs in several tumor types. Clinical studies demonstrated meaningful intracranial responses in patients with melanoma and non-small cell lung cancer (NSCLC) BM treated with ICIs,^[9–11]^ However, the benefit is not universal across all tumor types, and no phase III trials have yet confirmed robust clinical benefit for primary brain tumors such as gliomas.^[12,13]^

Dendritic cell (DC) based immunotherapeutic represents another promising approach for treating BMs. DCs are specialized antigen-presenting cells that play a crucial role in initiating and regulating adaptive immune responses.^[14,15]^ In DC-based vaccination, autologous DCs are loaded with tumor antigens ex vivo before being reintroduced to the patient, thereby potentially inducing tumor-specific immune responses that can target both intracranial and extracranial disease. This approach offers several potential advantages: it may overcome the limitations of the BBB by activating immune cells that can subsequently infiltrate brain tumors; it can generate memory T-cell responses that may provide long-term tumor control; and it often has a favorable toxicity profile compared to other systemic therapies.

Several clinical trials have evaluated DC vaccines in patients with various cancer types, including breast, colon, and lung cancers, with encouraging results.^[16–18]^ However, data specifically on patients with BMs are limited, creating a significant knowledge gap regarding the efficacy and safety of DC vaccination in this high-risk population.

In September 2018, Taiwan approved the revised “Regulations Governing the Application or Use of Specific Medical Techniques or Examinations, or Medical Devices” (henceforth referred to as the “Regulations of Special Medical Techniques”). This regulatory update facilitated the implementation of cell therapy programs in clinical settings, providing an opportunity to evaluate DC vaccination in real-world practice. While randomized controlled trials remain the gold standard for evaluating therapeutic efficacy, real-world data are increasingly recognized as valuable complements that can provide insights into treatment outcomes in heterogeneous patient populations encountered in routine clinical practice.

In this study, we present preliminary real-world results from a single-center experience with DC vaccination in patients with BMs who had undergone surgical resection. Our objective was to evaluate the safety profile and potential survival benefit of this approach in a diverse cohort of patients with BMs from various primary tumors. While the sample size and heterogeneous nature of our cohort limit definitive conclusions, these data may generate hypotheses and inform the design of future prospective studies of DC vaccination for this challenging patient population.

## 2. Methods

### 2.1. Patients and study design

This retrospective, single-institution analysis evaluated 17 patients with pathologically confirmed BMs who received conventional therapies along with autologous DC vaccination between January 2019 and December 2022 at China Medical University Hospital. The study was approved by the Institutional Review Board of China Medical University Hospitals (approval no. CMUH112-REC3-155). All procedures were conducted in accordance with the ethical standards of the institutional research committee and with the 1964 Helsinki Declaration and its later amendments. Written informed consent was obtained from all patients prior to treatment and data publication.

Eligible patients met the following criteria: age ≥ 18 years; pathologically confirmed BM from solid tumors or lymphoma; successful surgical resection of at least 1 BM lesion; Karnofsky Performance Scale (KPS) score ≥ 70; and adequate organ function to undergo apheresis and receive vaccination. Exclusion criteria included: pregnancy or breastfeeding; severe immunological compromise (absolute lymphocyte count < 500 cells/μL, active autoimmune disease requiring systemic immunosuppression); severe comorbidities with life expectancy < 3 months independent of cancer; and inability to provide informed consent.

## 3. Data collection and endpoints

We systematically collected the following data from electronic medical records: demographic information (age, sex); primary cancer characteristics (type, staging, response to prior therapies according to Response Evaluation Criteria in Solid Tumors [RECIST] v1.1); BM features (number, location, sites of initial dissemination); presence of extracranial metastases; treatments before and after BM diagnosis (surgery, radiotherapy, chemotherapy, targeted therapy); DC vaccination details (number of doses, timing, complications); and survival outcomes.

The primary endpoint was overall survival (OS), measured from the date of BM surgical resection to the date of death from any cause or last follow-up (censored data). Secondary endpoints included safety (assessed by monitoring for local and systemic adverse events at each vaccination visit and during follow-up appointments) and analysis of prognostic factors associated with survival outcomes.

To provide context for our results, we calculated the expected survival for each patient using the Diagnosis-Specific Graded Prognostic Assessment (DS-GPA) or Graded Prognostic Assessment (GPA) based on their primary tumor type, following established methodologies. This allowed comparison between observed survival in our cohort and expected survival based on validated prognostic indices.

## 4. Vaccine preparation and administration protocol

Fresh tumor samples were collected during surgical resection of BMs and processed according to a standardized protocol. After removing necrotic tissues and blood clots, specimens were placed in 4°C Hank balanced salt solution culture medium. The samples were mechanically dissociated and then enzymatically digested with a mixture containing 40 mg type IV collagenase and 100 U type V hyaluronidase in 40 mL of Hank balanced salt solution at room temperature for 3 hours.

The digested material was filtered through a 100-mesh nylon filter and washed twice with phosphate-buffered saline solution. The resulting tumor cell suspension was mixed with autologous patient serum in modified Eagle medium and cultured at 37°C in a 5% CO_2_ atmosphere. Cultured tumor cells underwent at least 3 passages before cryopreservation to ensure adequate expansion.

Approximately 2 months after surgery, peripheral blood mononuclear cells were obtained from each patient through standard leukapheresis procedures. CD14 + monocytes were isolated and cultured for 7 days in the presence of granulocyte-macrophage colony-stimulating factor (GM-CSF, 800 U/mL) and interleukin-4 (IL-4, 500 U/mL) to generate immature DCs. DC maturation was then induced by adding tumor necrosis factor-alpha (TNF-α, 50 ng/mL) for an additional 48 hours.

The resultant DCs and lymphocytes were examined for cell markers, including MHC class I and II, CD80, CD83, and CD86, using fluorescence-activated cell sorting and flow cytometry with monoclonal antibodies to confirm proper DC maturation. Quality control measures included assessment of DC viability (>80% required), sterility testing, and endotoxin testing (<5 EU/mL required).

Cultured tumor cells were inactivated by exposure to a 100 Gy radiation dose from a ^137^Cs source. Mature DCs and inactivated tumor cells were then co-cultured in a 1:1 ratio under 5% CO_2_ for 18 to 24 hours to allow antigen loading. Subsequently, the tumor antigen-loaded DCs were harvested, washed, and portioned into 10 doses of 1 mL, each containing 2 × 10^7^ DCs. Vaccine preparations were cryopreserved in liquid nitrogen until use.

All vaccines were administered according to a standardized protocol: weekly for the first 4 weeks, biweekly for the next 4 weeks, and monthly thereafter for up to 6 months (maximum 10 doses). Each dose was administered via bilateral subcutaneous injections into the axillary regions. Patients were monitored for 1 hour post-injection for immediate adverse events, and underwent regular clinical assessments (including vital signs, injection site examination, and symptom review) at each visit.

## 5. Monitoring and follow-up

Treatment response and disease status were assessed by brain magnetic resonance imaging (MRI) at baseline (postsurgery, prevaccination) and every 3 months thereafter. Additional imaging was performed if clinically indicated. Systemic disease was evaluated according to standard protocols for the primary tumor type, typically every 3 months or as clinically indicated. In selected cases, monitoring was supplemented with positron emission tomography or single-photon emission computed tomography.

Adverse events were systematically monitored and graded according to the Common Terminology Criteria for Adverse Events (CTCAE) version 5.0. Evaluation included clinical assessment, complete blood counts, and comprehensive metabolic panels at each vaccination visit and during follow-up appointments.

## 6. Statistical analysis

All statistical analyses were performed using MedCalc® Statistical Software version 23.0.5 (MedCalc Software Ltd, Ostend, Belgium). Descriptive statistics were used to characterize the patient population and treatment details. Categorical variables were reported as frequencies and percentages, while continuous variables were presented as means with standard deviations or medians with ranges, as appropriate.

Survival analyses were performed using the Kaplan–Meier method, with median survival time (MST) reported with 95% confidence intervals (CI). Comparisons between subgroups were conducted using the log-rank test. A *P*-value < .05 was considered statistically significant, though we acknowledge the exploratory nature of these analyses given the sample size limitations.

To address potential confounding factors, we conducted an exploratory analysis comparing subgroups based on: number of vaccine doses received (<6 vs ≥6); primary tumor type; and receipt of targeted therapy (for patients with lung cancer). Given the small sample size and retrospective nature of this study, these subgroup analyses are considered hypothesis-generating rather than definitive.

The sample size in this preliminary analysis was determined by the number of eligible patients treated at our institution during the study period rather than formal power calculations.

## 7. Results

### 7.1. Patient characteristics

Seventeen patients with surgically resected BMs who met the eligibility criteria received DC vaccines between January 2019 and December 2022. The median follow-up duration was 18 months (range: 3–51 months). Baseline demographic and clinical characteristics are summarized in Table 1.

**Table 1:** Baseline demographic and clinical characteristics of patients with brain metastasis treated with dendritic cell vaccination. Comprehensive presentation of patient characteristics including age, sex, primary cancer type, disease status at brain metastasis diagnosis, metastatic pattern, and surgical details.

No.	Age	Sex	Cancer type	RECIST at BM	SITE OF first dissemination	Extra cranial metastasis	No. of BM tumors	No. of BM resected	Surgical sites
1	35	M	NSCLC, adenocarcinoma	Newly diagnosed	Bone	Yes	1	1	Left parietal
2	48	F	Colon, sigmoid	Newly diagnosed	Liver	Yes	3	2	Right frontal, right parietal
3	78	M	Prostate	SD	Bone	Yes	3	1	Bilateral frontal
4	70	M	NSCLC, adenocarcinoma	SD	Bone	No	1	1	Left occipital
5	74	F	NSCLC, adenocarcinoma	PD	Bone	No	1	1	Left occipital
6	73	F	Esophageal	PR	Neck	Yes	2	2	Left cerebellum, right frontal
7	65	M	Lymphoma	Newly diagnosed	N/A	No	1	1	Right parietal
8	53	M	Nasopharyngeal cancer	Newly diagnosed	Brain	Yes	2	1	Left petrous
9	42	M	NSCLC, adenosquamous carcinoma	SD	Bone	Yes	1	1	Right frontal
10	64	F	NSCLC, adenocarcinoma	Newly diagnosed	Brain	Yes	1	1	Right frontal
11	48	F	Melanoma	SD	Bone	No	2	2	Right parietal, left frontal
12	39	F	Breast	SD	Brain	Yes	1	1	Left parietal
13	62	F	NSCLC, adenocarcinoma	SD	Pleural	Yes	3	1	Left occipital
14	59	F	Melanoma	PD	Bone	Yes	3	2	Left temporal, left frontal
15	43	M	RCC	SD	Brain	No	1	1	Right temporal
16	70	M	NSCLC, adenocarcinoma	SD	Brain	No	1	1	Left cerebellum
17	54	M	NSCLC, adenocarcinoma	SD	Pleural	Yes	1	1	Right cerebellum

BM = brain metastasis, F = female, M = male, NSCLC = non-small cell lung cancer, PD = progressive disease, PR = partial response, RCC = renal cell carcinoma, RECIST = Response Evaluation Criteria in Solid Tumors, SD = stable disease.

The study population had a mean age of 56.2 years (range: 35–78) with a balanced gender distribution (9 women, 8 men). The cohort exhibited diversity in primary malignancies: 8 patients (47.1%) had non-small-cell lung cancer (NSCLC), 2 (11.8%) had melanoma, and the remaining 7 patients (41.2%) had various other primary cancers (breast, colon, esophageal, nasopharyngeal, prostate, renal cell carcinoma, and lymphoma).

Five patients (29.4%) presented with BM as the initial manifestation of their cancer (newly diagnosed), while the remaining 12 patients (70.6%) developed BM during the course of their disease. Among patients with previously diagnosed cancer, 10 (83.3%) had stable disease or partial response to prior therapy per RECIST criteria, while 2 (16.7%) had progressive disease at the time of BM diagnosis. All previously diagnosed patients had received systemic therapy before BM detection, primarily consisting of chemotherapy or targeted agents, and 6 patients (50%) had previously undergone radiotherapy.

The distribution of metastatic disease showed that 11 patients (64.7%) had extracranial metastases in addition to BM. Common sites of initial dissemination included bone (n = 7, 41.2%), brain (n = 5, 29.4%), and pleural/pulmonary sites (n = 2, 11.8%). The median number of BMs per patient was 1 (mean = 1.65, range: 1–3), and the median number of surgically resected lesions was 1 (mean = 1.24, range: 1–2). The frontal lobe was the most common location for resected metastases (n = 7, 41.2%), followed by the occipital lobe (n = 3, 17.6%) and cerebellum (n = 3, 17.6%).

## 8. Treatment characteristics

Following surgical resection, 12 patients (70.6%) received adjuvant radiotherapy to the surgical cavity and/or remaining intracranial disease. Sixteen patients (94.1%) received systemic therapy after BM resection, including chemotherapy (n = 5), targeted therapy (n = 9), or combination approaches (n = 2). One patient (5.9%) received palliative care only due to rapid progression of primary cancer.

With respect to DC vaccination, the median time from surgery to first vaccine dose was 10 weeks (range: 8–14 weeks). Eleven patients (64.7%) received ≥ 6 doses of the vaccine (median: 7, range: 6–10), while 6 patients (35.3%) received < 6 doses (median: 3, range: 3–5). Reasons for receiving fewer than planned doses included: disease progression requiring alternative therapy (n = 3), patient preference (n = 2), and intercurrent illness unrelated to vaccination (n = 1).

## 9. Safety and tolerability

The DC vaccination was generally well-tolerated, with no grade 3–4 toxicities observed. The most common adverse events were mild (grade 1) injection site reactions, including erythema (n = 8, 47.1%), pruritus (n = 6, 35.3%), and pain (n = 5, 29.4%), all of which resolved spontaneously within 24–48 hours without specific intervention. Low-grade fever (37.5–38.0°C) was reported in 3 patients (17.6%) within 24 hours postvaccination and resolved with or without antipyretics. No systemic allergic reactions, autoimmune phenomena, or vaccine-related serious adverse events were observed throughout the treatment period.

One patient experienced postoperative wound healing complications following BM resection (prior to vaccination) and required surgical debridement. This event was deemed unrelated to the DC vaccine, as it occurred before vaccination was initiated.

## 10. Survival outcomes

**Figure 1.**
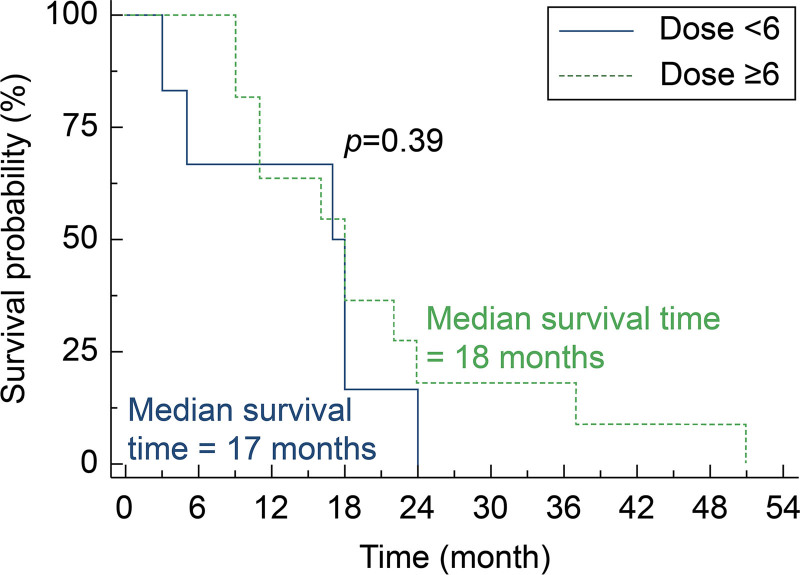
Kaplan–Meier survival curves stratified by number of dendritic cell vaccine doses received. Kaplan–Meier survival analysis comparing overall survival between patients who received > 6 doses versus ≤ 6 doses of dendritic cell (DC) vaccine after BM resection. MST was 18 mo for > 6 doses (95% CI: 9–24) and 17 mo for ≤ 6 doses (95% CI: 3–24); log-rank *P* = .39. CI = confidence interval, DC = dendritic cell, MST = median survival time.

At the time of analysis, 11 patients (64.7%) had died. The median overall survival (OS) for the entire cohort was 18 months (95% CI: 9–22 months) from the time of BM resection (Fig. 1). This compares favorably to the historical median OS of 14.5 months reported for patients undergoing surgical resection of BMs without DC vaccination.

We analyzed survival outcomes in relation to specific patient and treatment factors. Patients who received ≥ 6 doses of the vaccine demonstrated a median OS of 18 months (95% CI: 9–24 months) compared to 17 months (95% CI: 3–24 months) for those who received < 6 doses (log-rank test, *P* = .39; Figure 1). While this difference was not statistically significant, possibly due to the small sample size, it suggests a potential benefit from completing more vaccine doses.

We compared observed survival with expected survival based on validated prognostic indices. Of the 14 patients with available DS-GPA or GPA scores, 9 (64.3%) survived longer than their predicted MST based on these indices (Table 2), suggesting a potential benefit of the combinatorial approach including DC vaccination.

**Table 2:** Treatment characteristics and survival outcomes of patients with brain metastasis treated with dendritic cell vaccination. Detailed presentation of treatment regimens before and after brain metastasis resection, DC vaccine doses administered, prognostic scores, and survival outcomes.

No.	Vaccine dose	Estimated MST (mo)	Survival time after operation (mo)	ds-GPA/GPA index	Treatment before operation		Treatment after operation	
					Chemotherapy/target therapy	Radiation therapy	Chemotherapy/target therapy	Radiation therapy
1	10	30	51	2.5	Nil		Carboplatin + Pemetrexed + bevacizumab	Yes
2	4	3.4	18	1	Nil		Bevacizumab + iorinotecan + leucovorin + fluorouracil	Yes
3	8	N/A	11	1	Degarelix (3rd line)	Yes	Abiraterone	Yes
4	7	15	37	2	Gefitinib	No	Gefitinib	No
5	3	15	24	2	Docetaxel (3rd line)		Loratinib	No
6	5	6.5	5	1.5	Cisplatin	Yes	No (palliative)	
7	9	N/A	24	3	Nil		Rituximab + methotrexate + leucovorin	No
8	10	N/A	22	2.5	Nil		Gemcitabine + cisplatin	Yes
9	6	5	16	2	Afatinib	Yes	Osimertinib	Yes
10	6	15	18	2	Nil		Afatinib	Yes
11	7	15.8	18	3	Nivolumab	No	Trametinib + Dabrafenib	Yes
12	3	12.9	18	2	Goserelin acetate + letrozole	Yes	Palbociclib + goserelin acetate + anastrozole	Yes
13	3	15	17	1.5	N/A, previously managed in another facility		Erlotinib	Yes
14	3	8.3	3	1.5	Cyclophosphamide + methotrexate (2nd line)		Cyclophosphamide + methotrexate	No
15	10	17	11	3	Nil		Sunitinib	Yes
16	7	15	9	1.5	Docetaxel + pembrolizumab	Yes	Gemcitabine	Yes
17	6	15	9	1.5	Docetaxel (3rd line)	Yes	Docetaxel	Yes

DS-GPA = diagnosis-specific graded prognostic assessment, GPA = graded prognostic assessment, KPS = Karnofsky Performance Scale, MST = median survival time, N/A = not applicable.

Among the 14 patients with available DS-GPA/GPA scores, survival outcomes varied according to prognostic category: those with favorable scores (≥2.5, n = 3) achieved a median OS of 22 months (95% CI: 11–51 months), patients with intermediate scores (1.5–2.0, n = 7) had a median OS of 17 months (95% CI: 9–24 months), and those with poor scores (≤1.0, n = 4) demonstrated a median OS of 11 months (95% CI: 5–18 months).

Subgroup analysis of the 8 NSCLC patients revealed a median OS of 17.5 months (95% CI: 9–37 months). Given the heterogeneity of cancer types within our sample, identifying a single appropriate historical reference median OS for such a mixed cohort from published literature was challenging. As lung cancer represented the largest subgroup in our cohort, we utilized the median OS reported specifically for lung cancer BM patients undergoing surgery without DC vaccination in a comparable published study,^[19]^ as the reference value for our primary analysis, with one-sample Wilcoxon Signed-Rank test. There is significant longer median OS compared to the same 11.5 months reference value derived from the literature for lung cancer patients (*P* = .0391). This finding suggests the potential of DC vaccination as adjuvant therapy for in NSCLC patients with BM. Analyses for other cancer type subgroups were not performed due to sample sizes of <5 patients.

**Figure 2.**
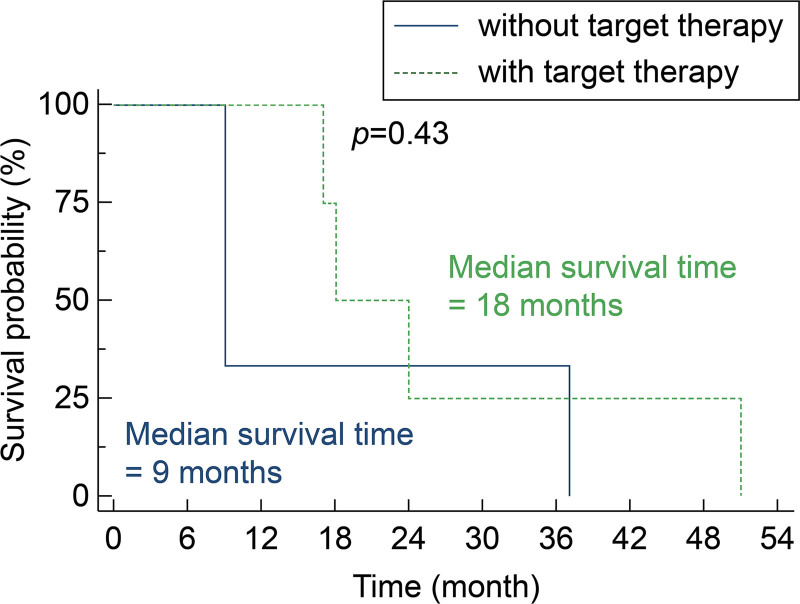
Kaplan–Meier survival curves of non-small cell lung cancer patients with brain metastasis stratified by receipt of targeted therapy. Overall survival from the time of brain metastasis resection in non-small cell lung cancer patients, comparing those who received targeted therapy (dashed green line) versus those who did not (solid blue line). Median survival time was 18 mo (95% CI: 17–51) for patients receiving targeted therapy compared to 9 mo (95% CI: 9–37) for those not receiving targeted therapy (Log-rank test, *P* = .43). Vertical tick marks indicate censored observations. CI = confidence interval.

Notably, the 5 NSCLC patients who received targeted therapy – based on actionable mutations or expressions such as EGFR, ALK, or PD-L1, as detailed in Table 3 – had a median overall survival (OS) of 18 months (95% CI: 17–51), compared to 9 months (95% CI: 9–37) for those who did not receive targeted therapy (log-rank test, *P* = .43; Figure 2). Although not reaching statistical significance, this finding suggests potential synergy between targeted therapy and DC vaccination in NSCLC patients with BM.

**Table 3:** Molecular characteristics and targeted therapy status of non-small cell lung cancer patients with brain metastasis. Summary of molecular profiles, targeted therapy administration, and initial performance status for the non-small cell lung cancer subgroup.

No	Cancer type	Target therapy	Initial KPS	EGFR	ALK	PD-L1 (>50%)
1	Adenocarcinoma	Yes	90	-	-	+
4	Adenocarcinoma	No	80	n/a	n/a	n/a
5	Adenocarcinoma	Yes	70	n/a	+	n/a
9	Adenosquamous carcinoma	Yes	70	+	n/a	n/a
10	Adenocarcinoma	Yes	80	+	n/a	n/a
13	Adenocarcinoma	Yes	50	+	n/a	n/a
16	Adenocarcinoma	No	70	-	-	n/a
17	Adenocarcinoma	No	80	-	-	-

ALK = anaplastic lymphoma kinase, EGFR = epidermal growth factor receptor, KPS = Karnofsky Performance Scale, MST = median survival time, n/a = not available, PD-L1 = programmed death-ligand 1.

## 11. Case illustration

**Figure 3.**
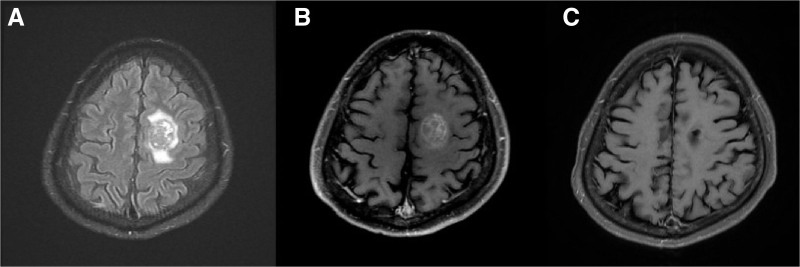
Representative magnetic resonance images of patient with long-term response to dendritic cell vaccination. Brain MRI images of Patient 1, a 36-yr-old man with brain metastasis from lung adenocarcinoma. (A) Preoperative T2 FLAIR image showing left parietal tumor with surrounding edema. (B) Preoperative T1 contrast-enhanced image demonstrating enhancing left parietal lesion. (C) T1 contrast-enhanced image 22 mo postsurgical resection and completion of dendritic cell vaccination, showing no evidence of tumor recurrence. This patient survived 51 mo from diagnosis despite having stage IV disease. FLAIR = fluid-attenuated inversion recovery, MRI = magnetic resonance imaging.

Patient 1, a 36-year-old man with no prior cancer history, presented with progressive right-sided weakness. Brain MRI revealed a left parietal tumor with peripheral edema (Fig. 3A and 3B). The patient underwent complete surgical resection, and pathology confirmed metastatic adenocarcinoma. Subsequent evaluation identified a primary lung adenocarcinoma (T1bN0M1c, and stage IVB) that was PD-L1 positive but EGFR and ALK negative.

Following surgery, the patient received carboplatin/pemetrexed/bevacizumab and adjuvant radiotherapy to the surgical cavity. DC vaccination was initiated 10 weeks postsurgery, and he completed all 10 planned doses without significant adverse events. Follow-up brain MRI 22 months after surgical resection showed no evidence of intracranial recurrence (Fig. 3C), although the primary disease eventually progressed. The patient subsequently received multiple lines of immunotherapy (pembrolizumab, nivolumab/ipilimumab, atezolizumab) for systemic disease management and survived for 51 months after BM diagnosis – significantly longer than the median expected survival of 30 months based on his DS-GPA score of 2.5.

This case illustrates the potential for long-term intracranial disease control with DC vaccination as part of a multimodal treatment approach, even in the context of systemic disease progression requiring subsequent therapies.

## 12. Discussion

### 12.1. Mechanism of action and therapeutic rationale

DCs function as critical mediators in the “cross-section” phenomenon, a fundamental component of effective antitumor immune response. As specialized antigen-presenting cells, DCs are uniquely positioned to capture, process, and present tumor antigens to both CD8 + and CD4+T cells, thereby initiating and coordinating adaptive immune responses.^[20,21]^ Intracellular antigens are presented to cytotoxic CD8 + T cells via major histocompatibility complex (MHC) class I molecules, while extracellular antigens are presented to CD4 + T helper cells via MHC class II molecules. This dual presentation capability makes DCs particularly valuable for targeting heterogeneous tumors like BMs.

In DC-based immunotherapy, the therapeutic approach involves several key steps: collection of autologous monocytes via apheresis; ex vivo differentiation and maturation of these monocytes into DCs; loading of DCs with tumor antigens derived from the patient’s own tumor tissue; and reintroduction of these tumor antigen-loaded DCs to stimulate antitumor immune responses. The resulting immune activation can potentially target microscopic residual disease after surgical resection, address both local and distant tumor sites, and establish immunological memory for long-term tumor surveillance.^[22,23]^

Unlike many systemic therapies, DC vaccination offers several theoretical advantages for BM treatment. First, activated T cells can cross the BBB, potentially overcoming a major limitation of conventional chemotherapy.^[24]^ Second, the specificity of the immune response may reduce collateral damage to normal brain tissue compared to whole-brain radiotherapy.^[25]^ Third, the potential for generating immune memory may provide ongoing surveillance against microscopic disease or recurrence.^[26]^ These advantages, coupled with the generally favorable toxicity profile observed in DC vaccination studies, make this approach particularly promising for patients with BMs who often face limited therapeutic options.

## 13. Integration with current evidence

While the evidence base for DC vaccination in BMs remains limited, our findings should be viewed in the context of existing literature on immunotherapy for CNS malignancies. The notion that the CNS is “immune-privileged” has evolved substantially, with mounting evidence demonstrating immune cell trafficking across the BBB, particularly in the context of malignancy.^[9,10]^ This evolving understanding has paved the way for immunotherapeutic approaches including both checkpoint inhibitors and cellular therapies like DC vaccination.

Several case reports and small series have previously demonstrated encouraging responses to DC vaccination in patients with BMs. Laurell et al^[27]^ reported a complete response in a patient with metastatic renal cell carcinoma who had both brain and liver metastases following allogeneic DC treatment. Similarly, Karbach et al^[28]^ documented a patient with melanoma-associated BMs who maintained complete remission for 10 years after treatment with radiosurgery and autologous tumor lysate-loaded DCs. Our cohort extends these observations by providing real-world data across multiple tumor types in a larger, albeit still limited, patient series.

The MST of 18 months observed in our study compares favorably with historical data reporting a median OS of 14.5 months for patients undergoing surgical resection of BMs without DC vaccination.^[8]^ Moreover, 64.3% of our patients with available DS-GPA/GPA scores survived longer than their predicted MST based on these validated prognostic indices. While these findings must be interpreted cautiously given the lack of a contemporaneous control group, they suggest a potential survival benefit that warrants further investigation in controlled trials.

Our results also align with previous studies of DC vaccination in specific tumor types. For instance, Takahashi et al^[29]^ conducted a multicenter retrospective analysis of 260 patients with advanced NSCLC treated with DC vaccination alone and reported a MST of 13.8 months from initial DC dose. In our subgroup of NSCLC patients with BMs, we observed a median OS of 18 months despite the traditionally poor prognosis associated with CNS involvement. This observation is particularly encouraging given that BMs often portend worse outcomes in lung cancer patients.

## 14. Subgroup analyses and potential predictive factors

Although our sample size limits definitive conclusions, several interesting patterns emerged from our exploratory subgroup analyses. First, patients who received 6 or more vaccine doses showed a trend toward improved survival (MST 18 months) compared to those receiving fewer doses (MST 17 months), although this difference did not reach statistical significance (*P* = .39). This trend aligns with immunological principles suggesting that repeated antigen exposure may be necessary to generate robust and durable immune responses. The observation that patients completing more vaccine doses tended to have better outcomes suggests that early integration of DC vaccination into treatment plans and strategies to ensure treatment completion may be important for optimizing outcomes.

Second, among NSCLC patients, the median OS is longer compared with recent study having similar patient group to our patients. Despite limitation of sample size, the statistically significant findings observed suggest a potential survival benefit associated with the DC vaccine, particularly evident in the NSCLC subgroup where the comparison is most direct. Also, those receiving targeted therapies alongside DC vaccination demonstrated a notably longer median survival (18 months) compared to those without targeted therapy (9 months). Although not statistically significant due to small numbers (*P* = .43), this finding raises the intriguing possibility of synergistic effects between molecularly targeted agents and immunotherapy. Emerging preclinical evidence suggests that certain targeted therapies may enhance antigen presentation, promote immunogenic cell death, or favorably modulate the tumor microenvironment.^[30]^ Our observations support the hypothesis that combining precision oncology approaches with immunotherapy may yield superior outcomes than either approach alone, particularly in molecularly selected patient populations.

The case of Patient 1 provides a compelling illustration of the potential benefits of multimodal therapy including DC vaccination. This patient, presenting with symptomatic BM as the initial manifestation of PD-L1-positive lung adenocarcinoma, demonstrated no intracranial recurrence during 22 months of follow-up after completing DC vaccination, despite eventual progression of systemic disease. His overall survival of 51 months significantly exceeded the expected 30 months based on prognostic factors, highlighting the potential for durable intracranial disease control with this approach.

## 15. Safety profile and clinical implementation

A key strength of DC vaccination appears to be its favorable safety profile. In our cohort, no severe (grade 3–4) adverse events attributable to vaccination were observed. Reported side effects were predominantly mild, transient injection site reactions and low-grade fever, consistent with previous studies of DC-based immunotherapy.^[31–33]^ This favorable toxicity profile is particularly valuable in patients with BMs, who often have limited physiological reserves and may be vulnerable to adverse effects of aggressive therapies.

The absence of significant adverse events, coupled with the outpatient administration protocol, suggests that DC vaccination could be readily integrated into multimodal treatment approaches without substantially increasing treatment burden or compromising quality of life. This contrasts with some systemic therapies that may require inpatient administration or carry risks of serious toxicities necessitating close monitoring or hospitalization.

## 16. Practical considerations and limitations

Despite these encouraging findings, several practical challenges must be acknowledged in implementing DC-based immunotherapy. First, the process requires collection and culture of sufficient viable tumor tissue, necessitating surgical resection rather than needle biopsy. This requirement naturally selects for patients with surgically accessible lesions and may exclude those with unresectable or numerous small metastases. Second, the approximately 2-month interval between surgery and vaccination – needed for tumor culture and DC preparation – introduces a delay that may be problematic for patients with aggressive disease. Finally, the complex manufacturing process requires specialized facilities and expertise, potentially limiting widespread implementation outside of centers with established cell therapy programs.

Our study has several important limitations that must be considered when interpreting the results. First, as a retrospective, single-arm study, it lacks a contemporaneous control group receiving standard therapy without DC vaccination. While we attempted to mitigate this by comparing observed survival with expected survival based on validated prognostic indices, this approach cannot fully account for all potential confounding factors. Second, our sample size of 17 patients limits statistical power and the ability to perform robust multivariable analyses. Third, the heterogeneity of primary tumor types in our cohort, while reflecting real-world practice, introduces variability that complicates interpretation. Fourth, all patients received multimodal therapy, making it challenging to isolate the specific contribution of DC vaccination to the observed outcomes.

Despite these limitations, our study provides valuable real-world data on DC vaccination for BMs across multiple tumor types. The observed safety profile and encouraging survival outcomes, particularly compared to historical data and prognostic expectations, support further investigation of this approach in larger, controlled studies.

## 17. Future directions

Based on our findings and the broader literature, several promising directions for future research emerge. First, prospective randomized trials specifically focused on BMs from individual tumor types (particularly NSCLC, melanoma, and breast cancer) would provide more definitive evidence regarding efficacy. Such trials should incorporate standardized manufacturing protocols, clearly defined dosing regimens, and comprehensive immune monitoring to elucidate mechanisms of response and resistance.

Second, the potential synergy between DC vaccination and targeted therapies or ICIs warrants systematic exploration. Combinations of these approaches might leverage complementary mechanisms of action: DC vaccination to prime tumor-specific immune responses and checkpoint inhibitors to prevent subsequent T-cell exhaustion. Preclinical models and early-phase clinical trials exploring these combinations with correlative studies would be particularly valuable.

Third, optimization of DC manufacturing protocols to reduce production time and enhance immunogenicity represents an important area for innovation. Approaches such as electroporation of tumor-derived mRNA, use of neoantigens identified through next-generation sequencing, or incorporation of adjuvants might further enhance the efficacy of DC-based approaches.

Finally, identification of predictive biomarkers for response to DC vaccination would enable better patient selection and more personalized application of this resource-intensive therapy. Comprehensive immune profiling of the tumor microenvironment, peripheral blood monitoring, and radiographic response assessment using novel imaging techniques may provide insights into which patients are most likely to benefit.

## 18. Conclusions

Our real-world experience with DC vaccination following surgical resection of BMs demonstrates that this approach is safe and feasible in clinical practice. The observed median survival of 18 months, which exceeds historical benchmarks and prognostic expectations in many cases, suggests potential therapeutic benefit. Although the nonrandomized nature of our study and the small sample size preclude definitive efficacy conclusions, our findings provide a rationale for larger, controlled studies of DC vaccination in this challenging patient population.

The trend toward improved outcomes in patients completing more vaccine doses suggests the importance of early treatment initiation and completion of the full vaccination course. Additionally, the particularly encouraging results observed in NSCLC patients receiving targeted therapies alongside DC vaccination highlight the potential for synergistic combinations of precision medicine and immunotherapy approaches.

Despite inherent limitations in study design, our results represent a meaningful contribution to the evolving evidence base for immunotherapy in BMs. These findings may help inform the design of future trials and guide clinical decision-making for patients with limited therapeutic options. As our understanding of CNS immunology and tumor-immune interactions continues to advance, DC vaccination holds promise as a component of multimodal strategies to improve outcomes for patients with BMs.

## Acknowledgments

We would like to thank Associate Professor Chia-Ing Li from the School of Medicine, China Medical University, for providing valuable guidance and assistance with the statistical analysis in this study.

## Author contributions

**Conceptualization:** Hung-Lin Lin, Der-Yang Cho, Chun-Chung Chen.

**Data curation:** Chih-Hsiu Tu, Wei-Lin Hsu, Yu-Chung Juan, Jeng-Hung Guo.

**Formal analysis:** Chun-Jen Chang, XianXiu Chen.

**Investigation:** Yu-Han Huang, Jeng-Hung Guo.

**Resources:** Wen-Liang Huang, Ming-Chao Liu.

**Supervision:** Der-Yang Cho, Chun-Chung Chen.

**Writing – original draft:** Chih-Hsiu Tu, Huai-Ping Ho.

**Writing – review & editing:** Hung-Lin Lin, Chun-Chung Chen.
